# Distributions of photosynthetic traits, shoot growth, and anti-herbivory defence within a canopy of *Quercus serrata* in different soil nutrient conditions

**DOI:** 10.1038/s41598-021-93910-5

**Published:** 2021-07-14

**Authors:** Masanari Norisada, Takeshi Izuta, Makoto Watanabe

**Affiliations:** 1 Graduate School of Agriculture, Tokyo University of Agriculture and Technology, Fuchu, Tokyo 183-8509 Japan; 2 Institute of Agriculture, Tokyo University of Agriculture and Technology, Fuchu, Tokyo 183-8509 Japan

**Keywords:** Ecology, Plant sciences, Ecology

## Abstract

The hypothesis of the present study is that not only distributions of leaf photosynthetic traits and shoot growth along light gradient within a canopy of forest trees, but also that of leaf anti-herbivory defence capacities are influenced by soil nutrient condition. To test this hypothesis, we investigated the distributions of photosynthetic traits, shoot growth, anti-herbivory defence and leaf herbivory rate throughout the canopy of *Quercus serrata* grown in two sites with different soil nutrient conditions. In both sites, photosynthetic traits, shoot growth, and anti-herbivory defence were greater in the upper canopy. The overall defence and herbivory rate in the lower nutrient condition were higher and lower than those in the higher nutrient condition, respectively. Although differences in leaf traits between upper and lower canopies in the higher nutrient condition were smaller than those in the lower nutrient condition, no difference was found for anti-herbivory defence. These results suggest that soil nutrient condition does not affect the distributions of leaf anti herbivory defence along light gradient within a canopy of *Q. serrata*.

## Introduction

Light intensity in a developed canopy of trees decreases from top to bottom. Leaf traits and shoot growth acclimate to their light condition for efficient light utilisation in whole-canopy photosynthesis^1–4^. For example, sun leaves in upper canopy have higher leaf mass per area (LMA) and nitrogen allocation to Calvin cycle enzymes (mainly Rubisco), while shade leaves in lower canopy have lower LMA and higher chlorophyll content to minimize light limitation of photosynthesis^1,5,6^. Chemical defence capacities against herbivore also show a distribution within a canopy^7,8^.

Several studies revealed that distributions of leaf traits are influenced by soil nutrient condition. Niinemets et al.^9,10^ reported responses of needle morphological traits and concentrations of nitrogen and phosphorous to light gradient within a canopy of *Pinus sylvestris* was lower at the nutrient poor site than at the nutrient rich site. In these studies, needle nitrogen and phosphorous contents were correlated with photosynthetic capacities, indicating distribution of photosynthetic capacity is also influenced by soil nutrient condition. Ishii et al.^11^ showed similar trend in the morphological traits of *Picea jezoensis* needles in the comparison of site with well-developed brown forest soil and that with immature volcanic ash soil, whereas there was no different response in *Picea glehnii* needles.

Many studies indicate soil nutrient condition influences defence capacity against herbivore^12–14^. Generally, plants grown in high nutrient condition have lower defence capacity, and vice versa^15,16^. However, there was no study on the effects of soil nutrient condition on the distribution of defence capacities within a canopy. Leaf morphological traits such as leaf density and thickness affect defence against herbivore^17^. Defence chemicals are produced from photosynthate^5^. Both morphological and photosynthetic traits distributions within a canopy are influenced by soil nutrient condition as mentioned above^9–11^. In this context, distributions of defence capacities would be influenced by soil nutrient condition.

The hypothesis of the present study is that not only distributions of leaf photosynthetic traits and shoot growth along light gradient within a canopy, but also that of leaf traits on anti-herbivory defence capacity are influenced by soil nutrient condition. To test this hypothesis, we investigated the distributions of these leaf traits and herbivory rate in leaves within the canopy of mature *Quercus serrata* trees grown under different soil nutrient conditions.

## Results

The mean air temperature and soil volumetric water content (VWC) were higher in Field Museum (FM) Tamakyuryo than in FM Chichibu (Table 1). The tree height, diameter of the breast height (DBH), and leaf area index (LAI) in FM Tamakyuryo were also greater than those in FM Chichibu. The concentration of inorganic nitrogen (NH_4_^+^-N, NO_3_^−^-N  and total of them) in the soil of FM Tamakyuryo was higher than that in the soil of FM Chichibu although the difference of NO_3_^−^-N was not significant. There was no significant difference in concentrations of total nitrogen and available phosphorus and pH of the soil between the two sites.

**Table 1:** Mean air temperature and volume water content from April to September 2018, the height, diameter of the breast height (DBH) and the leaf area index (LAI) of *Q. serrata* grown in FM Tamakyuryo and FM Chichibu in 2018, the concentrations of inorganic nitrogen (NH_4_^+^-N, ﻿NO_3_^−^-N and total of them) and available phosphorus, and pH in the soils of FM Tamakyuryo and FM Chichibu 2018. Temperature and VWC is mean of 1 h average value from April to September 2018. Height, DBH, LAI, NH_4_^+^-N, NO_3_^−^-N, total nitrogen, available phosphorus and pH are mean value of nine individuals or soils.

	Tamakyuryo	Chichibu	ANOVA
Temperature (℃)	21.2 (4.1)	17.8 (3.5)	n.s
VWC (%)	30.2 (2.3)	25.7 (3.7)	***
Height (m)	21.6 (1.0)	14.6 (0.4)	***
DBH (cm)	39.4 (3.7)	17.5 (1.2)	***
LAI	3.9 (0.5)	2.5 (0.0)	***
NH_4_^+^-N (mg g^−1^)	0.057 (0.022)	0.031 (0.016)	**
NO_3_^−^-N (mg g^−1^)	0.12 (0.11)	0.06 (0.02)	n.s
Total inorganic N (mg g^−1^)	0.177 (0.091)	0.094 (0.029)	*
N (%)	0.41 (0.02)	0.48 (0.05)	0.071
P (mg kg^−1^)	6.9 (1.9)	11.2 (5.2)	n.s
pH	5.80 (0.12)	5.91 (0.04)	n.s

Each value is mean value and values in parentheses indicate standard deviation.

One-way ANOVA: *p < 0.05, ***p < 0.001, n.s. not significant. Actual *p* value is shown if 0.05 ≤ *p* < 0.10.

**Figure 1:**
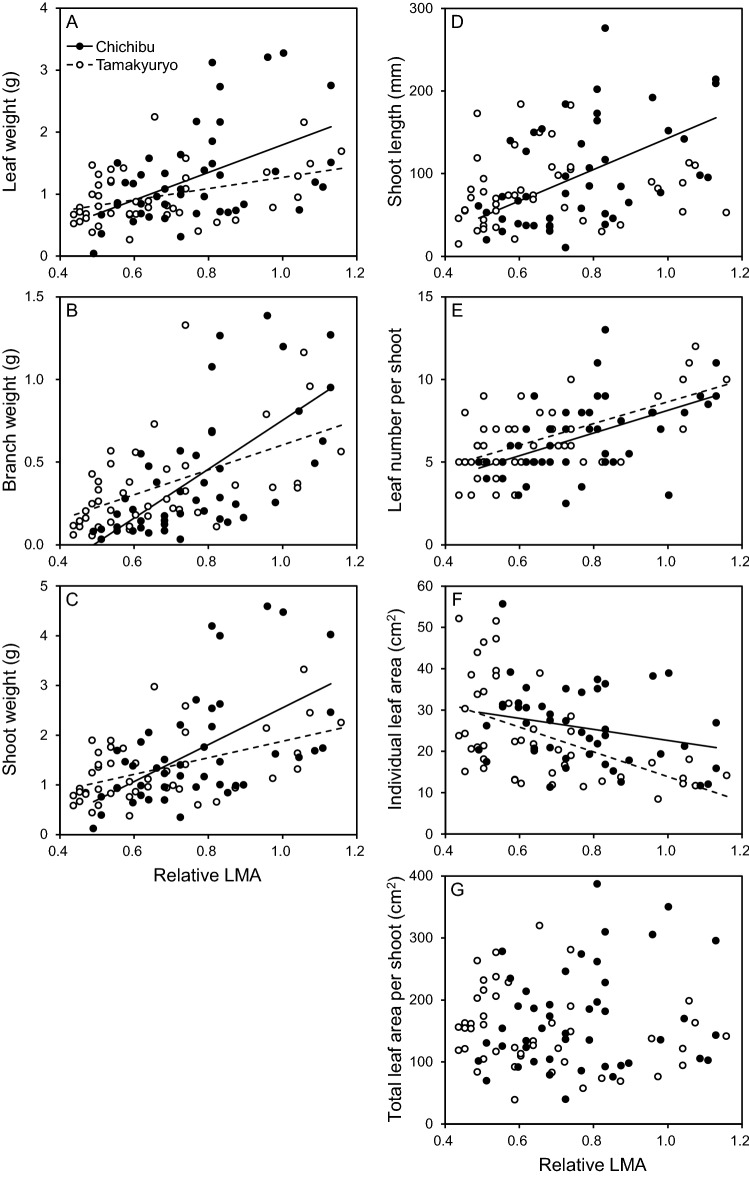
Dry mass of (**a**) leaf, (**b**) branch, (**c**) shoot, (**d**) shoot length, (**e**) leaf number per shoot, (**f**) individual leaf area, and (**g**) total leaf area per shoot as a function of relative LMA of *Q. serrata* grown in FM Tamakyuryo in July 2018 (while circle) and FM Chichibu in August 2018 (black circle) (n = 45). Dotted and solid lines indicate regression line of FM Tamakyuryo and FM Chichibu, respectively. Regression lines were drawn when there was a significant effect of relative LMA.

All parameters, except for individual leaf area and total leaf area per shoot, significantly increased with the increase in relative LMA (*p* < 0.001, Table 2, Fig. 1). The slopes of the regression lines between relative LMA and leaf weight, branch weight, or shoot weight in FM Tamakyuryo were significantly gentler than those in FM Chichibu (*p* < 0.05, Table 2, Fig. 1A–C). In FM Chichibu, shoot length increased with an increase in relative LMA, while the regression line in FM Tamakyuryo was not significant (Fig. 1D). The total leaf area per shoot was affected neither by relative LMA nor site although there was significant interaction of them. (Fig. 1G).

**Table 2:** Result of analysis of deviance for the effect of site and relative LMA on leaf weight, branch weight, shoot weight, shoot length, leaf number per shoot, individual leaf area, total leaf area per shoot, Rubisco, chlorophyll, total phenolics, condensed tannin, leaf density, leaf thickness, herbivory rate, *N*_mass_ and *P*_mass_ of *Q. serrata* grown in FM Tamakyuryo in July 2018 and FM Chichibu in August 2018.

	Site	Relative LMA	Site × relative LMA
Leaf weight	n.s	***	*
Branch weight	n.s	***	*
Shoot weight	n.s	***	*
Shoot length	n.s	**	**
Leaf number per shoot	n.s	***	n.s
Individual leaf area	n.s	***	n.s
Total leaf area per shoot	n.s	n.s	*
Rubisco	n.s	***	0.054
Chlorophyll	n.s	***	*
Total phenolics	0.082	***	n.s
Condensed tannin	**	***	n.s
Leaf density	**	***	n.s
Leaf thickness	***	***	**
Herbivory tare	***	n.s	n.s
*N*_mass_	n.s	***	n.s
*P*_mass_	*	0.051	***

*p < 0.05, **p < 0.01, ***p < 0.001, n.s. not significant. Actual *p* value is shown if 0.05 ≤ *p* < 0.10.

**Figure 2:**
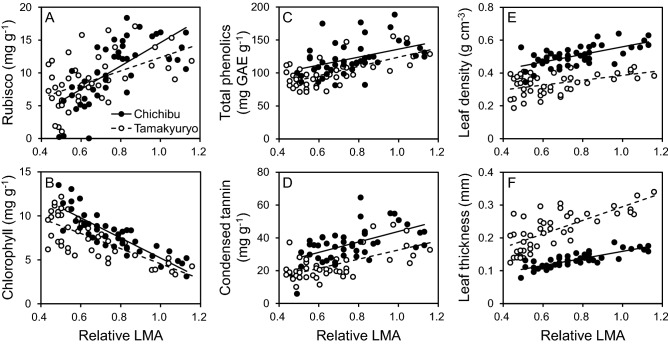
Concentration of (**a**) Rubisco, (**b**) chlorophyll, (**c**) total phenolics, and (**d**) condensed tannin; (**e**) leaf density; and (**f**) leaf thickness as a function of relative LMA of leaves of *Q. serrata* grown in FM Tamakyuryo in July 2018 (while circle) and FM Chichibu in August 2018 (black circle) (n = 45, except Rubisco: n = 44). Dotted and solid lines indicate regression line of FM Tamakyuryo and FM Chichibu, respectively. Regression lines were drawn when there was a significant effect of relative LMA. Total phenolics concentration is expressed as mg of gallic acid equivalent per g.

The concentrations of Rubisco, total phenolics, condensed tannin, leaf density, and leaf thickness were significantly increased with increasing relative LMA (*p* < 0.001), whereas the opposite trend was observed in chlorophyll concentration (*p* < 0.001, Fig. 2). The slopes of the regression lines between relative LMA and Rubisco or chlorophyll concentration were different between the sites and those in FM Tamakyuryo were moderate compared with those in FM Chichibu although the difference for Rubisco was marginal (*p* = 0.054, Table 2, Fig. 2A,B). The concentration of condensed tannin and leaf density in FM Tamakyuryo were significantly lower than those in FM Chichibu throughout the canopy (*p* < 0.001, Table 2, Fig. 2C,E). We observed similar but marginal trend in total phenolics concentration (*p* = 0.082, Table 2, Fig. 2D). There was no significant interaction of the site and relative LMA on the concentrations of total phenolics, condensed tannin, and leaf density, and the regression lines were almost parallel between the sites (Table 2). In contrast, throughout the canopy, a higher leaf thickness was observed in FM Tamakyuryo than in FM Chichibu (Fig. 2F). Furthermore, the slope of the regression line was significantly steeper in FM Tamakyuryo than in FM Chichibu (Table 2).

**Figure 3:**
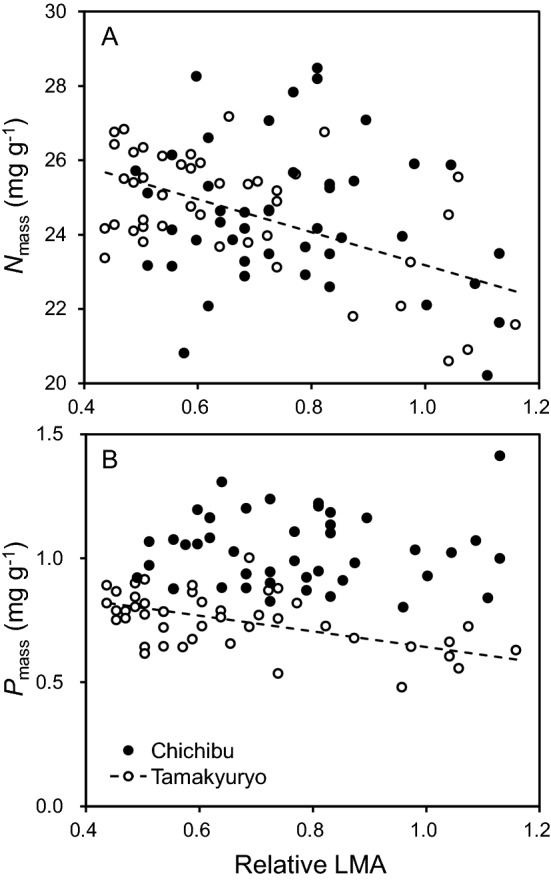
Concentration of (**a**) nitrogen and (**b**) phosphorus per unit leaf dry mass (*N*_mass_ and *P*_mass_, respectively) as a function of relative LMA of *Q. serrata* grown in FM Tamakyuryo in July 2018 (while circle) and FM Chichibu in August 2018 (black circle) (n = 45). Dotted line indicates regression line of FM Tamakyuryo. Regression line was drawn when there was a significant effect of relative LMA.

The mass-based nitrogen and phosphorus concentration (*N*_mass_ and *P*_mass_, respectively) in the leaves in FM Tamakyuryo significantly decreased with increasing relative LMA (Table 2, Fig. 3). In FM Chichibu, *N*_mass_ and *P*_mass_ did not significantly respond to relative LMA. The *P*_mass_ was significantly greater in FM Chichibu than in FM Tamakyuryo throughout the canopy (Table 2).

**Figure 4:**
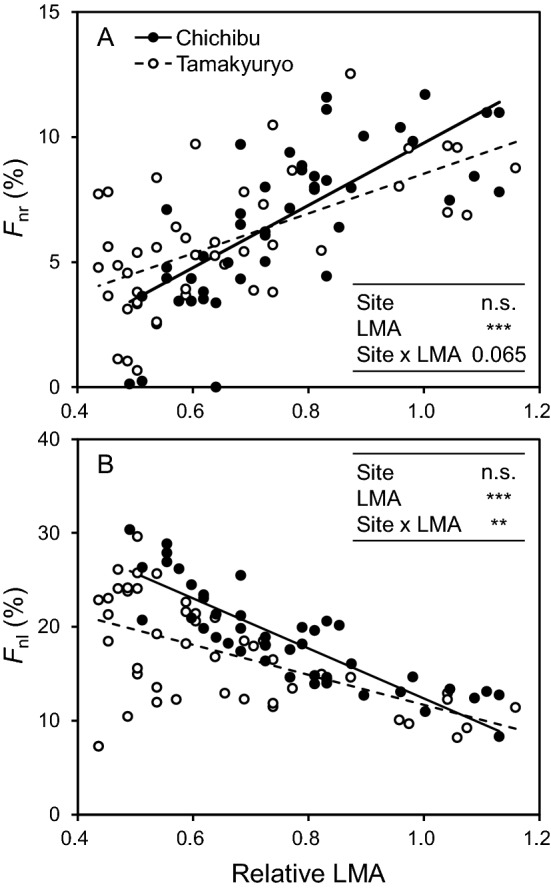
Fraction of leaf (**a**) nitrogen in Rubisco (*F*_nr_) and (**b**) light harvesting complex and photosystems (*F*_nl_) as a function of relative LMA of *Q. serrata* grown in FM Tamakyuryo (while circle) and FM Chichibu in 2018 (black circle) (n = 44). Dotted and solid lines indicate regression line of FM Tamakyuryo and FM Chichibu, respectively. Regression lines were drawn when the effect of relative LMA was significant. **p < 0.01, ***p < 0.001, n.s., not significant. Actual *p* value is shown if 0.05 ≤ *p* < 0.10.

In both sites, fraction of leaf nitrogen in Rubisco (*F*_nr_) was increased as the relative LMA increased, whereas the opposite trend was observed in light-harvesting complex and photosystems (*F*_nl_) (Fig. 4). The slopes of the regression lines between relative LMA and *F*_nr_ or *F*_nl_ were different between the sites, while the significance was marginal in *F*_nr_ (*p* = 0.065). Similar to those for Rubisco and chlorophyll, the slopes were moderate in FM Tamakyuryo compared with those in FM Chichibu.

**Figure 5:**
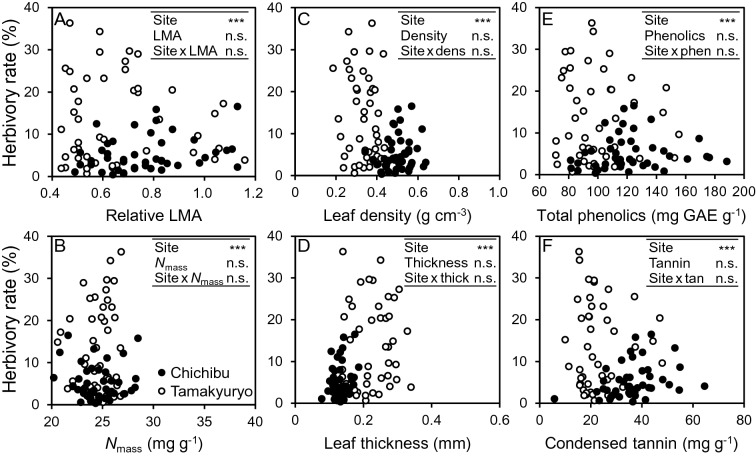
The value of herbivory rate as a function of (**a**) relative LMA, (**b**) *N*_mass_, (**c**) leaf density, (**d**) leaf thickness, (**e**) total phenolics, and (**f**) condensed tannin of *Q. serrata* grown in FM Tamakyuryo in July 2018 (while circle) and FM Chichibu in August 2018 (black circle) (n = 45). *p < 0.05, ***p < 0.001, n.s., not significant.

Figure 5 shows the herbivory rate in the leaves of *Q. serrata* as a function of several leaf parameters. In all explanatory variables, the herbivory rate was greater in FM Tamakyuryo than in FM Chichibu (*p* < 0.001). Average herbivory rate in FM Tamakyuryo and FM Chichibu were 13.7% and 5.2%, respectively. Neither the explanatory variables alone nor the interaction of site and explanatory variables affected the herbivory rate.

## Discussion

In both sites, the Rubisco concentration in leaves of *Q. serrata* were greater in the upper canopy (Fig. 2A), whereas leaf chlorophyll concentration was greater in the lower canopy (Fig. 2B). These distributions were explained by the difference of nitrogen allocation to them (Fig. 4). Our results, from mature *Q. serrata*, support the previous studies reported the distributions of Rubisco and chlorophyll concentrations and nitrogen allocation to them within a canopy^6,18–20^. Shoot growth was also greater in the upper canopy (Fig. 1). This could be owing to the higher production of photosynthate with higher light intensity in upper canopy leaves^5,21^.

Total phenolics and condensed tannin concentration was higher in the upper canopy leaves as compared to the lower canopy leaves (Fig. 2C, D). This result also matches with the previous studies^7,21^. High light intensity and abundant photosynthate would promote the activity of phenylalanine ammonium lyase (PAL), which is a key enzyme related to secondary metabolism^22,23^. A higher activity of PAL results in an increase in the production of defensive chemicals against leaf herbivory^7,24^.

Leaf density, which affects physical defences to leaf herbivory, was also greater in the upper canopy (Fig. 2E). Tanaka et al*.*^25^ observed a higher leaf density in upper canopy leaves, where light intensity is higher, in mature dipterocarp trees. Leaves under a high light intensity develop thicker and denser palisade parenchyma than those under low light intensity^26^. Although the reason for this enhanced leaf density at a higher irradiance is not clear, the greater production of the palisade parenchyma in the limited space between the epidermis of leaves might lead to the high leaf density^26^.

Based on the distributions of chemical and physical defence capacities, we expected that leaf herbivory would be restricted to the upper canopy in both sites. However, there was no significant difference in the leaf herbivory rate among the canopy positions (Fig. 5A). Various tendencies of leaf herbivory rates within a canopy have been reported. Yamasaki & Kikuzawa^8^ showed a greater herbivory rate in the lower canopy leaves of mature *Fagus crenata*. In contrast, Rowe & Potter^27^ observed a greater herbivory rate in the leaves in the upper canopy of *Tilia cordata*. Le Corff & Marquis^28^ reported no difference in herbivore densities between the understory and canopy for *Q. alba* and *Q. velutina*. Jamieson et al*.*^29^ suggested that primary metabolites (e.g., non-structural carbohydrates) are also determining factors of leaf herbivory. Considering other leaf traits and herbivore activities throughout the canopy would enable us to consider the factors that determine the distribution of the leaf herbivory rate within a canopy of mature trees.

Generally, plants grown in high nutrient conditions develop lower defensive capacities than those grown in lower nutrient conditions^12–15^. Tripler et al*.*^30^ reported that leaf damage by herbivores increases in the saplings of nine tree species under fertile conditions. Kolstad et al*.*^16^ reported lower concentrations of defensive compounds in beech and spruce saplings grown in fertile conditions. In the present study, the lower chemical and physical defence capacities and higher average herbivory rate as the whole canopy were observed in *Q. serrata* grown in FM Tamakyuryo with higher nutrient condition as compared to FM Chichibu with lower nutrient condition (Table 2, Figs. 2 and 5). Although the defence capacities did not explain the distribution of herbivory rate within the canopy, the relation of defence capacities and herbivory rate as whole canopy level was in agreement with the previous studies.

The range in the values of shoot growth and concentrations of Rubisco and chlorophyll within a canopy of *Q. serrata* in FM Tamakyuryo was similar to that in FM Chichibu despite the difference in soil nutrient condition (Table 1, Fig. 2A,B), which indicates that the photosynthetic capacity of an individual leaf does not simply correspond to the soil nutrient condition. However, the LAI in FM Tamakyuryo was higher than that in FM Chichibu (Table 1). The photosynthetic rate is regulated not only by leaf capacity but also by environmental factors, such as light intensity, CO_2_ concentration, and hydraulic conditions^5^. Thus, there is a limitation in the enhancement of photosynthetic production by increasing leaf nitrogen content at the individual leaf level. Therefore, *Q. serrata* grown under fertile conditions in the present study would enhance whole-plant photosynthetic production by increasing LAI but not the nitrogen content in individual leaves.

The distributions of photosynthetic capacities and shoot growth along with the light gradient in the canopy were different between the two sites (Table 2; Figs. 1,2). However, the distribution of anti-herbivory defence capacities was not influenced by the soil nutrient conditions, indicating our hypothesis was rejected. These results suggest regulation mechanisms of canopy distributions in photosynthetic traits and shoot growth of *Q. serrata* differ with that of anti-herbivory defence capacities. On the other hand, there are many kinds of defence chemicals^5^ and the effects of soil nutrient condition on the distribution in a canopy may depend on each chemical. Future studies with detail analysis of defence chemicals will progress our understanding of the mechanism in the effects of soil nutrient conditions on the distribution of defence capacity along with the light gradient within a canopy.

It is great concern that increase of nitrogen availability in forest ecosystem due to increasing atmospheric deposition of reactive nitrogen since the Industrial Revolution^31^. Our study will contribute not only to understand of ecological traits of forest trees but also to prediction of the relationship between tree and herbivore in the future environment with enhanced nitrogen availability.

## Methods

### Study sites and research trees

The study sites were FM Tamakyuryo (35°4ʹN, 139°2ʹE, *ca.* 170 m a.s.l., Hachioji, Tokyo, Japan) and FM Chichibu (35°6′N, 138°5′E, *ca.* 760 m a.s.l., Chichibu, Saitama, Japan) of Tokyo University of Agriculture and Technology. In the collection of plant materials, we followed the guideline of domestic law and got permission from the management body, Field Science Center of Tokyo University of Agriculture and Technology. The amounts of nitrogen deposition were reported as 19.1 and 4.9 kg N ha^−1^ year^−1^ in FM Tamakyuryo and FM Chichibu, respectively^32,33^. We set three plots at each site and selected three *Q. serrata* individuals in each plot. The stand ages were about 70 years old in FM Tamakyuryo and 30 years old in FM Chichibu and the canopies in both sites were considered to be fully developed^34,35^. The LAI in both sites was measured using a leaf area index sensor (MIJ-15LAI Type II/K2, Environmental Measurement Japan Co, Ltd., Fukuoka, Japan) with the following Eq. (1), parameterised for *Q. serrata*^36^:

1 $${\text{LAI }} = {\text{ 2}}.{\text{8}}0{\text{ }}*{\text{ ln}}\left( {{\text{NiR }}/{\text{ PAR}}} \right){\text{ }} + {\text{ }}0.{\text{69}}$$

where NiR and PAR are near-infrared radiation (µmol m^−2^ s^−1^) and photosynthetic active radiation (µmol m^−2^ s^−1^), respectively. The measurement was carried out on cloudy days to avoid direct sunlight.

Air temperature and relative humidity were monitored at 30-min intervals using a TR-72U Thermo Recorder (T&D Corporation, Nagano, Japan) and a RS-13 Thermo Recorder (Espec Mic Corporation, Aichi, Japan) from April to September 2018. The VWC was measured at 1-h intervals using a 10HS Moisture Sensor (METER Group Inc., Pullman, WA, USA) from May to September 2018.

### Soil analysis

In FM Tamakyuryo and FM Chichibu, 10 cm deep soil was collected in July and August 2018, respectively. The soil was air-dried for 7 days, and stored in a polypropylene bottle until the measurements. The total nitrogen content was measured using a CN corder (MT-700, YANAKO, Tokyo, Japan). The standard curve was created using commercial hippuric acid (7.82% N). The ammonium ions (NH_4_^+^) and nitrate ions (NO_3_^−^) were determined using the indophenol blue colourimetric method^37^ and an ion chromatography (883 Basic IC Plus, Metrohm, Tokyo, Japan), respectively. Soil pH in water extract was measured with a pH metre (D-22, HORIBA, Tokyo, Japan). Available phosphorus in the soil was extracted using the Bray 2 method and measured using the molybdenum blue colourimetric method^38–40^.

### Shoot sampling and measurements of morphological traits and herbivory rate

In FM Tamakyuryo and FM Chichibu, current-year shoots, branches and leaves, were collected in July and August 2018, respectively. We employed a rope access technique for the access to the canopy of target trees. We collected shoot samples from five different heights in the canopy. After the shoots sampling, leaf disks for biochemical analyses and LMA measurement were immediately collected. Leaf disks for biochemical analyses were frozen in dry ice. We determined thickness of leaf disks for LMA measurement and placed in an envelope. Then, the leaf disks and remaining shoots were brought back to the laboratory. The leaf disks for biochemical analyses and those for LMA measurement were stored at -80 °C and were dried in an oven at 80 °C for more than 3 days, respectively. The dried leaf disks were weighed for LMA calculation. Leaf density was calculated as LMA divided by leaf thickness.

Shoot length, base diameter of the branch, leaf number and leaf area were measured on the day of shoot sampling. The branches and leaves were separated in the border of the leaf blade and petiole. Leaves were scanned using a scanner (CanoScan LiDE 210 JP, Canon, Tokyo, Japan) and leaf area was calculated using image analysis software (Lia32, https://www.agr.nagoya-u.ac.jp/~shinkan/LIA32/). The branches and leaves were dried in an oven at 80 °C for more than 3 days and the dry mass was measured.

The leaf herbivory rate was measured using images acquired by scanning the leaves. We recreated the undamaged leaves using Paint software (version 6.3, Microsoft, WA, USA) by painting the area eaten by herbivores. Then, the leaf area was calculated again using the image analysis software. We calculated the herbivory rate using the following Eq. (2):

2 $${\text{Herbivory}}\;{\text{rate }}\left( \% \right) = \left( {LA_{{\text{1}}} {-}LA_{{\text{2}}} } \right){\text{/}}LA_{{\text{1}}} *{\text{1}}00$$

where *LA*_1_ is the undamaged leaf area that was recreated and *LA*_2_ is the leaf area of the sampled leaf (damaged leaves).

### Biochemical analyses

For the analysis of Rubisco, frozen leaf discs were powdered with liquid nitrogen using a pestle and mortar and homogenised with extraction buffer containing 100 mM HEPES–KOH (pH 8.0), 1 mM ethylenediaminetetraacetic acid, 0.7% (w/v) poly(ethylene glycol) 20,000, and 1% (w/v) Tween 80, as described previously^41^. The supernatant after centrifugation (16,000 g at 4 °C for 10 min) was use for the analysis. The Rubisco protein in the supernatant was separated by SDS-PAGE^42^. The gel was scanned with the scanner and the amount of Rubisco was calculated from the density of CBB-stained large subunit bands scanned using the image analysis software (ImageJ, http://rsb.info.nih.gov/ij/). A standard curve was made with bovine serum albumin. Chlorophyll was extracted with dimethyl sulfoxide according to Barnes et al*.*^43^, and determined using a spectrophotometer.

For the analysis of total phenolics and condensed tannin, the frozen leaf disks were freeze-dried (FDU-035, EYELA, Tokyo, Japan), and extracted with 50% methanol. The extract was heated at 40 °C for 60 min with ultrasonic vibration (SU-9TH, SIBATA, Saitama, Japan), and centrifuged at 16,000 g at 20 °C for 10 min. The supernatant was used as the sample solution. The concentration of total phenolics was determined by the Folin–Ciocalteu method as described by Julkunen-Tiitto^44^. The concentration of total phenolics was expressed as gallic acid equivalents (GAE). The concentration of condensed tannin was determined using the butanol-HCl method as described by Bate-Smith^45^. The condensed tannin concentration was calculated using the following Eq. (3):

3 $${\text{Condensed}}\;{\text{tannin}}\;{\text{concentration}}\left( {{\text{mg}}\;{\text{g}}^{{ - {\text{1}}}} } \right) = \left( {\left( {\left( {{\text{4}}.{\text{5 }}*A_{{{\text{55}}0}} } \right)/{\text{4 }} + 0.0{\text{11}}} \right)/{\text{2}}0.{\text{255}}} \right)*{\text{5}}000/M$$

where *A*_550_ is the absorbance at 550 nm and *M* is the dry mass of the leaf disc.

The leaf nitrogen content was measured using a CN corder. Leaf phosphorus was extracted using the wet ashing method^46^ with HNO_3_, HCl, and 30% hydrogen peroxide and determined using the molybdenum blue colourimetric method^39^. A standard curve was created using KH_2_PO_4_. The leaf nitrogen and phosphorus content were calculated as mg per g of leaf dry mass.

We calculated the *F*_nr_ and *F*_nl_. The *F*_nr_ was calculated assuming that the concentration of nitrogen in Rubisco is 16%^47,48^. The *F*_nl_ was calculated assuming that the nitrogen content per unit chlorophyll is 37.1 mol mol^−1^^49^.

### Evaluation of light condition with relative leaf mass per area

**Figure 6:**
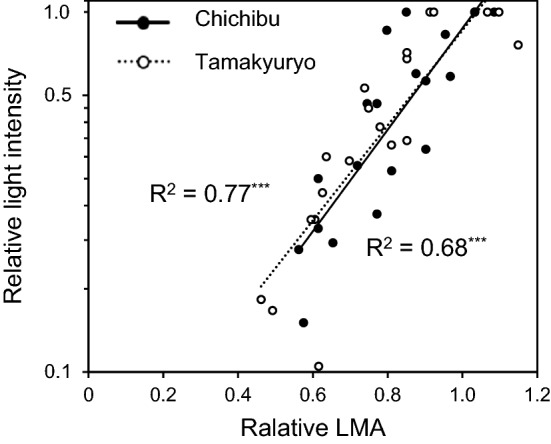
The relationship between relative LMA and relative light intensity in a canopy of *Q. serrata* grown in FM Tamakyuryo (while circle) and FM Chichibu in 2018 (black circle) (n = 20). Dotted and solid lines indicate regression line of FM Tamakyuryo and FM Chichibu, respectively. Y-axis was transformed into log-scale. In x-axis, higher relative LMA indicates upper canopy and lower relative LMA indicates lower canopy. ***p < 0.001.

Light intensity is one of the most important factors determining leaf traits at a given position within a canopy^8,50,51^. We used LMA as a parameter of relative light intensity within a canopy^52,53^. In addition to the above-mentioned main sampling, we collected shoot samples from five different heights in the canopy of four trees at each site and measured relative light intensity in each shoot position to the light intensity above the canopy using a GaAsP photodiode (Hamamatsu Photonics, Aichi, Japan). This measurement was carried out on cloudy days to avoid direct sunlight. Figure 6 shows the relationship between the relative LMA and relative light intensity at the two sites. The coefficients of determination for the regression lines were 0.77 (*p* < 0.001) and 0.68 (*p* < 0.001) in FM Tamakyuryo and FM Chichibu, respectively. In the main sampling, we calculated the mean LMA of leaves collected from the top of the nine trees at each site as reference LMA. The relative LMA of a given leaf sample was calculated as the ratio of LMA of the sample to the reference LMA at each site to standardise the difference in absolute LMA between the two sites.

### Statistical analysis

Statistical analyses were conducted using R software, version 3.5.1 [^54^, https://www.R-project.org]. The effects of site and relative LMA on leaf traits and leaf herbivory rate were analysed using a generalised linear mixed model with site and relative LMA as explanatory variances and plot as a random effect. Response variables, except for herbivory rate, were assumed to follow a Gaussian distribution, whereas the herbivory rate was assumed to follow a Gamma distribution. Then, we applied a type II analysis of deviance to test the single and combined effects of the explanatory variables^55^. Significant interaction of site and relative LMA means significant difference in slopes of the regression line of the response variance against relative LMA between two sites. We defined the effect of relative LMA as an index of the distribution of leaf traits and herbivory rate along with light gradient in canopy. We also checked the model with height instead of site (i.e. height and relative LMA) as explanatory factor. However, the fitting for data was better in the model with site than that with height in all variances. For temperature, VWC, tree height, DBH, LAI, NH_4_^+^-N, NO_3_^−^-N, total inorganic nitrogen, available phosphate, and pH, one-way ANOVA was used to test the effect of the site. In the analysis, a plot was nested within each site and added to the model as a random factor^56^.

## Data Availability

The datasets generated during and/or analysed during the current study are available from the corresponding author on reasonable request.
